# TRIM16 inhibits proliferation and migration through regulation of interferon beta 1 in melanoma cells

**DOI:** 10.18632/oncotarget.2466

**Published:** 2014-11-03

**Authors:** Selina K. Sutton, Jessica Koach, Owen Tan, Bing Liu, Daniel R. Carter, James S. Wilmott, Benafsha Yosufi, Lauren E. Haydu, Graham J. Mann, John F. Thompson, Georgina V. Long, Tao Liu, Grant McArthur, Xu Dong Zhang, Richard A. Scolyer, Belamy B. Cheung, Glenn M. Marshall

**Affiliations:** ^1^ Children's Cancer Institute, Lowy Cancer Research Centre, UNSW Australia, Sydney, Australia; ^2^ Melanoma Institute Australia and the University of Sydney, Sydney, Australia; ^3^ Tissue Pathology and Diagnostic Oncology, Royal Prince Alfred Hospital, Sydney and Discipline of Pathology, Sydney Medical School, The University of Sydney, Australia; ^4^ Molecular Oncology Laboratory, Oncogenic Signaling and Growth Control Program, Peter MacCallum Cancer Centre, East Melbourne, VIC 3002, Australia; ^5^ Sir Peter MacCallum Department of Oncology, The University of Melbourne, Parkville, VIC 3010, Australia; ^6^ Priority Research Centre for Cancer Research, Oncology and Immunology Unit, University of Newcastle, NSW, Australia; ^7^ Kids Cancer Centre, Sydney Children's Hospital, Sydney, Australia

**Keywords:** Melanoma, TRIM16, BRAF inhibitor, cell migration, IFNβ1

## Abstract

High basal or induced expression of the tripartite motif protein, TRIM16, leads to reduce cell growth and migration of neuroblastoma and skin squamous cell carcinoma cells. However, the role of TRIM16 in melanoma is currently unknown. TRIM16 protein levels were markedly reduced in human melanoma cell lines, compared with normal human epidermal melanocytes due to both DNA methylation and reduced protein stability. TRIM16 knockdown strongly increased cell migration in normal human epidermal melanocytes, while TRIM16 overexpression reduced cell migration and proliferation of melanoma cells in an interferon beta 1 (IFNβ1)-dependent manner. Chromatin immunoprecipitation assays revealed TRIM16 directly bound the IFNβ1 gene promoter. Low level TRIM16 expression in 91 melanoma patient samples, strongly correlated with lymph node metastasis, and, predicted poor patient prognosis in a separate cohort of 170 melanoma patients with lymph node metastasis. The BRAF inhibitor, vemurafenib, increased TRIM16 protein levels in melanoma cells *in vitro*, and induced growth arrest in BRAF-mutant melanoma cells in a TRIM16-dependent manner. High levels of TRIM16 in melanoma tissues from patients treated with Vemurafenib correlated with clinical response. Our data, for the first time, demonstrates TRIM16 is a marker of cell migration and metastasis, and a novel treatment target in melanoma.

## INTRODUCTION

Melanoma is an aggressive malignancy which is responsible for 80% of skin cancer deaths [1]. While surgical excision can cure localized disease, metastatic melanoma treated with conventional therapies has a median survival of only 6–9 months historically [2, 3]. Metastatic melanoma is highly resistant to traditional cytotoxic chemotherapies [3, 4]. New targeted melanoma treatments, such as BRAF inhibitors, vemurafenib (PLX4032) and dabrafenib (GSK2118436) [5, 6] offer higher patient response rates due to specific BRAF^V600^ targeting [4, 5]. However, disease progression is usually observed after a median of 5–7 months, highlighting the need for a better understanding of disease pathogenesis [7]. Combination of dabrafenib and trametinib is now the standard of care for BRAF^V600^ patients, with 50% of patients experiencing a progression free survival of 9–10 months [8].

The tripartite motif (TRIM) family of proteins are characterized by a RING B-box-coiled-coil protein domain architecture and have been implicated in the pathogenesis of multiple cancers, functioning as either oncogenes or tumor suppressors [9, 10]. TRIM16 has a role in innate immune function [11] and is secreted by keratinocytes in a caspase-1 dependent manner, a process enhanced by interleukin-1β (IL-1β). TRIM16 increased differentiation markers in keratinocytes and was highly expressed in basal keratinocytes, but was down-regulated during the proliferative phase of wound healing [12]. TRIM16 expression was significantly reduced *in vivo* during the progression from normal skin to squamous cell carcinoma (SCC), and inhibited SCC cell migration *in vitro* [13]. In addition, TRIM16 down-regulated protein-binding partners, cytoplasmic vimentin and nuclear E2F1, in neuroblastoma cells [14]. Together these data suggested that TRIM16 repressed cancer cell replication and migration. However, the role of TRIM16 in melanoma is unknown.

The regulatory inflammatory cytokine, interferon beta 1 (IFNβ1) has been used in the therapy of melanoma [15]. The human interferon gene region (containing Interferon-β1 (IFNβ1)) is deleted in numerous cancers, including melanoma [16, 17]. In melanoma, IFN-β gene transfer induced cell death *in vitro* [18–20] and suppressed melanoma cell proliferation [21, 22]. Both IFN-α2b and IFN-β1a inhibited tumor growth and lymph node metastasis in human melanoma xenografts [23]. Of all interferons, human IFNβ demonstrated the highest anti-proliferative activity against human melanoma cell lines [20]. In contrast to IFN-2α, IFN-β bound with higher affinity to the IFN α/β receptor 1 (IFNAR1), and induced a greater degree of apoptosis in melanoma cells [15]. IFNβ may also have some clinical activity in patients with resected high risk melanoma [24]. Transcription of IFN-β requires the formation of the multi-protein enhanceosome complex [25, 26]. The c-Jun/ATF-2 heterodimer was essential for the formation of the enhanceosome complex, and hence, IFN-β transcription [27].

We used human melanoma cell lines and excised human melanoma samples to show that loss of TRIM16 expression led to enhanced cell migration *in vitro*, and metastatic disease *in vivo*. The BRAF inhibitor, vemurafenib, suppressed melanoma cell growth in a TRIM16-dependent manner. Overexpression of TRIM16 in melanoma cells up-regulated IFNβ1 and c-Jun levels, which was required for the anti-proliferative TRIM16 effect. Lastly, low TRIM16 expression associated with poor prognosis in melanoma patients with lymph node metastasis, collectively indicating that TRIM16 represents a novel therapeutic target in melanoma.

## RESULTS

### TRIM16 protein expression is reduced in melanoma cell lines

**Figure 1:**
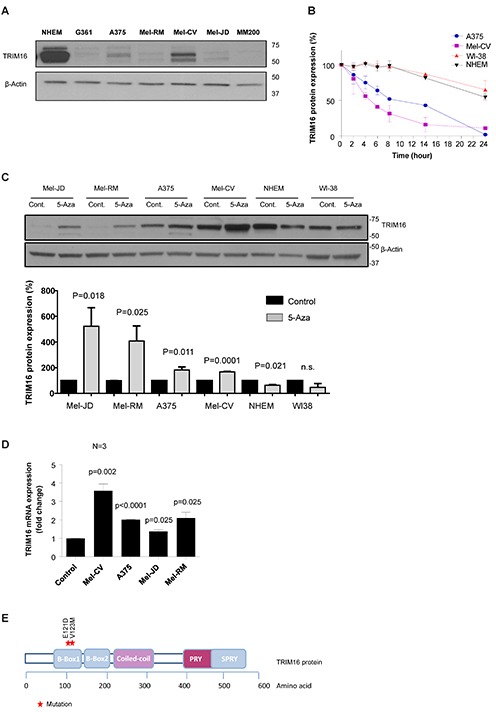
TRIM16 protein expression and half-life is decreased in melanoma cell lines compared to NHEM and is increased following treatment with the demethylating agent, 5-Aza **(A)** Immunoblotting analysis of TRIM16 protein expression in NHEMs and 6 melanoma cell lines. Whole cell lysates were prepared and Western blots were probed with an anti-TRIM16 antibody. The Western blot was also probed with anti-β-actin as a loading control. **(B)** TRIM16 protein half-life was analysed by Western blotting in melanoma cell lines (Mel-CV, A375), NHEM and WI-38 human fibroblasts following CHX treatment. Cells were treated with cycloheximide at a final concentration of 100 μg/ml over 24 hours. At the specified time points, the cells were harvested and total cellular protein was extracted for Western blotting. **(C)** Melanoma cell lines (Mel-JD, Mel-RM, A375, Mel-CV), NHEM and WI-38 human fibroblasts were treated with solvent control or 30 μM 5-Aza for 72 hours. In the top panel, the whole cell lysates were prepared and Western blots were probed with anti-TRIM16 and anti-actin antibodies. In the bottom panel, the TRIM16 protein expression in 5-Aza treated samples were quantified and represented as a percentage of solvent control. A statistically significant difference for TRIM16 expression level with and without 5-Aza is indicated. **(D)** TRIM16 mRNA expression was measured compared to solvent controls by RT-qPCR in melanoma cell lines, (Mel-CV, A375, Mel-JD, Mel-RM) treated with 30 μM 5-Aza for 24 hours. **(E)** Diagrammatic representation of the TRIM16 protein and individual domains, with sites of the two tumor related mutations.

We first analyzed the TRIM16 protein expression level by Western blotting in 6 melanoma cell lines, and showed a significantly lower level, compared to a normal human epidermal melanocyte cell line (NHEM) (Fig. 1A). The two protein bands at 67kDa and 64kDa on the Western blot using the TRIM16-specific antibody represent two isoforms of the TRIM16 protein caused by differential post-translational modification [12, 28]. The proteasome inhibitor MG-132 increased TRIM16 protein levels in melanoma cells (Supplementary Fig. 1A). The TRIM16 protein half-life in melanoma cell lines, A375 and Mel-CV, was 12 hours and 6 hours, respectively, compared with more than 24 hours in NHEM and normal human fibroblasts (WI38) (Fig. 1B and Supplementary Fig. 1B).

To determine whether loss of TRIM16 expression was also due to DNA methylation, we treated NHEM, WI38 and four melanoma cell lines with the demethylating agent, 5-aza-2′-deoxycytidine (5-Aza), at 30 μM for 72 hours. TRIM16 protein levels were significantly increased in the four melanoma cell lines following 5-Aza treatment (*P*=0.018 for Mel-JD, *P*=0.025 for Mel-RM, *P*=0.011 for A375 and *P*=0.0001 for Mel-CV), compared with normal cells (Fig. 1C). TRIM16 transcription also increased following 5-Aza treatment (Fig. 1D) in melanoma cell lines (*P*=0.05), indicating direct or indirect de-repression of the TRIM16 gene transcription upon treatment with the demethylating agent.

We next performed DNA sequencing of the endogenous TRIM16 coding and promoter regions for 9 melanoma cell lines. Melanoma cell lines IPC-298, MM200, A375, G361, SK-Mel-2, Mel-CV, Mel-JD, Mel-RM, and CHL-1 were sequenced. We found an E121D missense mutation in exon 1 of IPC-298, in the B-Box1 domain of the TRIM16 protein in 1/9 melanoma cell lines (Fig. 1E). The E121D mutation has also been reported in a publicly available database, catalogue of somatic mutations in cancer (COSMIC), in 1/71 stomach cancers [29]. In addition, a missense mutation at V123M was reported among 1/31 primary melanomas.[29] The B-box1 domain of TRIM protein, MID1, structurally resembles the ring domain of E3 ubiquitin ligases.[30] TRIM16 has a 46% sequence similarity with MID1, can adopt ring domain-like folds and has E3 ligase function *in vitro* and *in vivo* [31], indicating that mutation in TRIM16 B-box1 may perturb E3 ligase activity.

### TRIM16 overexpression reduces cell proliferation and migration of melanoma cells

**Figure 2:**
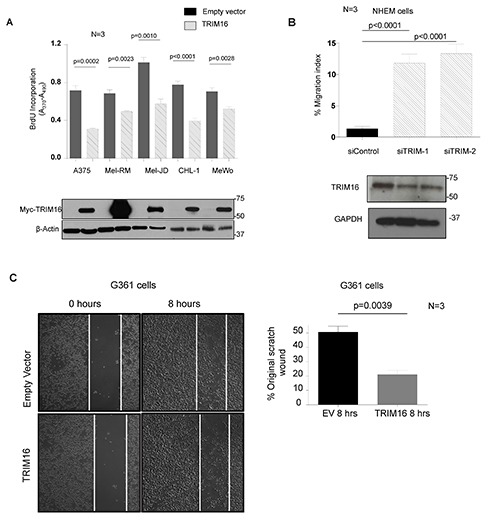
TRIM16 over-expression reduces melanoma cell proliferation and migration **(A)** A panel of melanoma cell lines were transiently transfected with TRIM16 plasmid DNA for 48 hours. Cell proliferation was measured by BrdU incorporation. The lower panel immunoblot confirmed TRIM16 transfection using an anti-Myc tag antibody. A statistically significant difference between empty vector (EV) and TRIM16-transfected cells is indicated. **(B)** NHEMs were transfected with scrambled siRNA control or two different TRIM16-specific siRNAs for 24 hours. The transwell migration assay was performed using conditioned media as a chemo-attractant. The histogram displays the percentage of the migrated cells divided by the total number of cells initially loaded into the wells. **(C)** representative phase contrast micrographs of the closure of scratch-wounded confluent cultures of melanoma cells (G-361) transfected with empty vector or TRIM16 plasmid DNA for 48 hours, photographed either immediately or 8 hours after wounding. The histogram displays the proportion of wound closure over 8 hours from the time of wounding.

To determine the effects of TRIM16 overexpression on melanoma cell growth, we transiently transfected 5 melanoma cell lines with the pcDNA3.1 empty vector or pcDNA3.1/TRIM16/myc.his expression vector. Overexpression of TRIM16 reduced cell proliferation for all 5 melanoma cell lines (Fig. 2A). We next examined the role of TRIM16 in melanoma cell migration. Knockdown of TRIM16 using TRIM16-specific siRNAs in the normal cellular counterpart of melanoma cells, NHEMs, resulted in a significant increase in cell migration (P<0.001) (Fig. 2B). Conversely, transient overexpression of TRIM16 for 48 hours in G361 melanoma cells caused a reduction in cell migration into a scratch wound *in vitro* over an 8 hour period (P<0.001) (Fig. 2C).

### TRIM16 directly binds to the IFNβ1 promoter and induces IFNβ1 transcription

**Figure 3:**
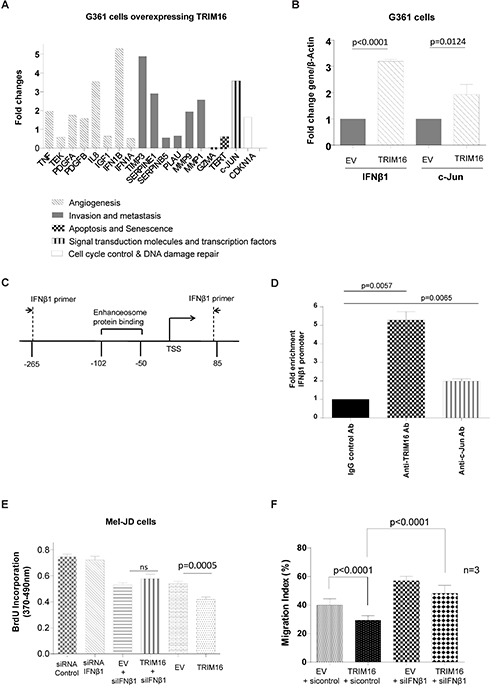
TRIM16 directly binds the *IFNβ1* gene promoter and induces *IFNβ1* transcription **(A)** Fold change in candidate gene mRNA expression as detected by RT-qPCR in melanoma cells (G-361) transiently transfected with a TRIM16 expression vector for 24 hours. Each gene is also annotated for categories of known function relevant to cancer. **(B)** mRNA expression of IFNβ1 and c-Jun in melanoma (A375, G361) cells by RT-qPCR 24 hours after transient transfection of either an EV or a TRIM16 expression vector. **(C)** A diagrammatic representation of the IFNβ1 gene promoter indicating transcription start site (TSS) and enhanceosome binding site is shown. A chromatin immunoprecipitation assay was performed using anti-TRIM16 or anti-c-Jun antibodies in the immunoprecipitation with IgG as a negative control. PCR was performed with IFNβ1 primers targeting the indicated regions of the IFNβ1 promoter region in G361 cells. **(D)** G361 cells were transfected with TRIM16 plasmid DNA for 48 hours, and the fold enrichment of the IFNβ1 promoter is shown for binding of TRIM16 and c-Jun antibodies and the IgG control to the IFNβ1 promoter. A control PCR 2000bp upstream of the IFNβ1 primer PCR site is used as a negative control. Fold enrichment of IFNβ1 gene core promoter by control, anti-TRIM16 or anti-c-Jun Ab was calculated by dividing PCR products from primers targeting the IFNβ1 gene core promoter by PCR products from primers targeting control region. Fold enrichment by control antibody was artificially set as 1.0. **(E)** Mel-JD cells were transiently transfected with either control siRNA, IFNβ1 siRNA, EV + siIFNβ1, TRIM16 plasmid DNA + siIFNβ1, EV, or TRIM16 plasmid DNA for 24 hours. Cell proliferation was measured by the BrdU incorporation assay in three independent experiments. **(F)** Invasion assay of G361 cells through collagen-coated cell culture inserts. The cells were transiently transfected with control siRNA + EV, TRIM16 plasmid DNA + control siRNA, EV + siIFNβ1, TRIM16 plasmid DNA + siIFNβ1 for 24 hours. The percentage of migrated cells divided by the total number of cells in the wells from three independent experiments is shown.

We have previously shown that TRIM16 can bind DNA, acetylate histones and enhance transcription of retinoid-responsive genes [32]. To investigate potential downstream target genes of TRIM16 in melanoma, we performed a Cancer Pathway PCR Array using mRNAs from G361 cells transiently transfected with empty vector or TRIM16 expression vector for 48 hours (Fig. 3A). The most highly induced gene in the expression array was IFNβ1 (5.2-fold). A number of other genes demonstrated more than a 2-fold increase, such as IL-8, TIMP3, SERPINE1, MMP1 and c-Jun. We confirmed by RT-qPCR that TRIM16 induced IFNβ1 and c-Jun mRNA expression in melanoma cells (Fig. 3B). To investigate whether TRIM16 directly bound the IFNβ1 promoter, we transiently transfected G361 cells with TRIM16 plasmid DNA for 48 hours, followed by a chromatin immunoprecipitation (ChIP) assay, and showed that both anti-TRIM16 and anti-c-Jun antibodies efficiently immunoprecipitated the region of the IFNβ1 gene core promoter carrying the enhanceosome protein binding site (Fig. 3C and 3D) [25]. To determine whether IFNβ1 expression was required for TRIM16-reduced cell proliferation, we transiently over-expressed TRIM16 in the presence of IFNβ1 siRNA knockdown and showed abrogation of the TRIM16-mediated growth inhibition (Fig. 3E). In addition, we transiently over-expressed TRIM16 in the presence of c-Jun siRNA knockdown and also showed abrogation of TRIM16-mediated growth inhibition (Supplementary Fig. 2). To determine whether IFNβ1 expression was required for TRIM16-reduced cell migration, we transiently over-expressed TRIM16 in the presence of IFNβ1 siRNA knockdown in G361 cells and showed that IFNβ1 was partially required for TRIM 16-mediated inhibition of cell migration (Fig. 3F).

### TRIM16 protein expression is reduced in metastatic melanoma and correlates with overall patient survival risk in stage III disease

**Figure 4:**
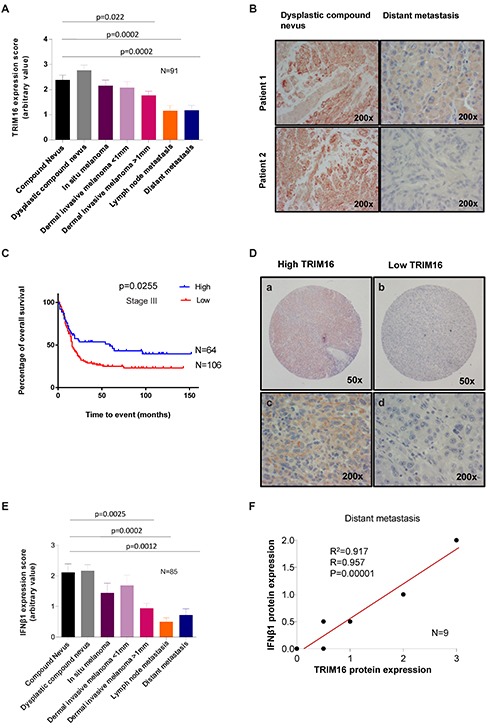
TRIM16 protein expression is reduced in metastatic melanoma and correlates with overall survival risk in stage III disease **(A)** The level of TRIM16 protein expression was analysed by immunohistochemistry by two observers blinded to clinical outcome using a polyclonal TRIM16 antibody and graded on an arbitrary scale of 0-4. The score of TRIM16 expression was collected from 91 patient samples with 13 samples per tumor type/ melanoma stage. The statistical comparisons were performed using student's t-test. A statistically significant difference for TRIM16 expression level between melanoma and compound nevi is indicated by p value. **(B)** Representative immunohistochemistry images of TRIM16 staining in two samples of dysplastic compound nevi and distant metastases. **(C)** Kaplan-Meier curve of overall patient survival for TRIM16 protein expression level, using the median value to subdivide the cohort for ‘high’ and ‘low’ staining. Patient median survival was 59 months for TRIM16 expression level of >1, and 16 months for TRIM16 expression level of <1. The data was analysed with the Log-rank test. **(D)** Representative immunohistochemical staining of lymph node metastases showing lymph node metastasis with high (a, c) or low (b, d) TRIM16 expression. **(E)** The level of IFNβ1 expression in primary and metastatic melanocytic tumors was measured for 85 melanoma patients using immunohistochemical grading after staining with an anti-IFNβ1 antibody. There were 13 samples per tumor type/stage for compound nevus to dermal invasive melanoma >1 mm and 11 samples for lymph node metastasis and 9 for distant metastasis. The statistical analysis was performed by the student's t-test. A statistically significant difference is indicated by **p<0.01 or ***p<0.001, when expression in melanoma was compared with compound nevi. **(F)** A correlation analysis between IFNβ1 and TRIM16 expression in distant melanoma metastases (n = 9).

We investigated the pattern of TRIM16 expression by immunohistochemistry (IHC) among 91 patient tumor samples, representing all clinical stages. A significant progressive reduction of TRIM16 protein expression level was found for dermally invasive primary melanoma, lymph node metastases and distant metastases, when compared to compound nevi (P<0.001) (Fig. 4A&B). The most significant decrease in TRIM16 expression was observed between the dermal invasive melanoma >1mm stage and the lymph node metastasis stage.

Therefore, we next determined the prognostic significance of low TRIM16 expression in an independent cohort of 170 melanoma patients with lymph node metastases (Stage III). Patients were subdivided into high or low expression subgroups based on the average value of 0.995. Low TRIM16 expression in melanoma cells metastatic to lymph nodes was significantly associated with poor prognosis [(Hazard ratio 0.6322 with 95% confidence of 0.4322 to 0.9383) vs (Hazard ratio 1.582 with 95% confidence of 1.066 to 2.314) (*P* = 0.0255, two-sided log-rank test; n = 170)] (Fig. 4C&D). Importantly, Stage III patients with a TRIM16 expression level >1 had a median survival of 59 months compared to patients with a TRIM16 expression level of <1 of only 16 months.

To further elucidate the role of IFNβ1 in melanoma progression, we performed IHC on the same 91 human patient samples analyzed for TRIM16 expression. We found that IFNβ1 protein expression levels were lower as melanoma became more invasive reaching significance at the dermal invasive melanoma >1mm thickness dermal invasiveness stage, *P*=0.0025 immediately before the disease becomes metastatic (Fig. 4E and Supplementary Fig. 3). The relationship between IFNβ1 expression and clinical stage closely mirrored the patterns seen for TRIM16 expression. Among a group of patients with distant metastatic disease, we observed a strong positive correlation between TRIM16 and IFNβ1 levels (Fig. 4F).

### TRIM16 protein is increased with vemurafenib treatment and is required for the drug action in melanoma cells

**Figure 5:**
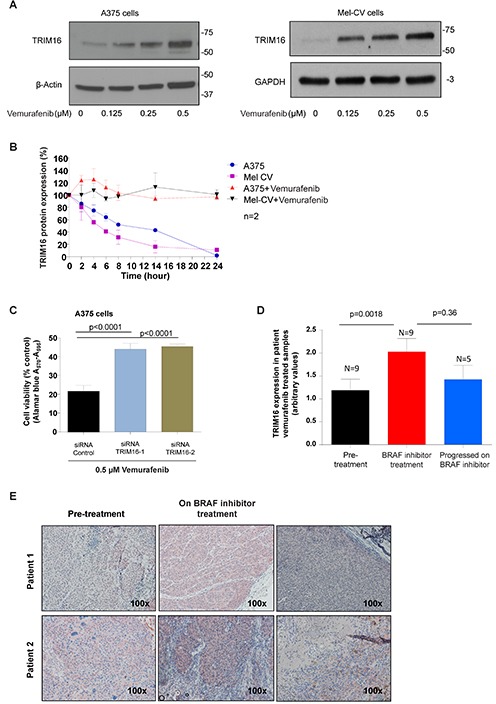
TRIM16 protein is increased with vemurafenib treatment and is required for the drug action **(A)** Melanoma (A375 and Mel-CV) cells were treated with vemurafenib at 0, 0.125, 0.25 or 0.5μM for 72 hours and whole cell lysates were used for immunoblotting against an anti-TRIM16 antibody. Anti-β-Actin was used as a loading control. **(B)** TRIM16 protein stability was assessed in melanoma (A375 and Mel-CV) cells following treatment with vemurafenib at 0.5 and 1.5μM respectively, and CHX at 100 μg/mL. At the specified time points, the cells were harvested and the protein was extracted for analysis by Western blotting against anti-TRIM16 or anti-β-actin. **(C)** Melanoma (A375) cells were transiently transfected with two different TRIM16 siRNA's or control siRNA, and then treated with 0.5 μM vemurafenib for 72 hours. Cell viability was measured using the Alamar blue assay. A statistically significant difference is indicated. **(D)** TRIM16 expression level was measured by immunohistochemistry in patient samples of pre-treatment (N=9), on vemurafenib (N=2) or dabrafenib (N=7) treatment, and progressed on BRAF inhibitor treatment (N=5). **(E)** Representative immunohistochemistry for two patients is shown.

To evaluate the possible therapeutic application of TRIM16 expression patterns on the treatment of melanoma, we investigated the effect of the BRAF inhibitor, vemurafenib, on TRIM16 expression levels. After 72 hours of vemurafenib treatment at doses of 0–0.5 μM in A375 and Mel-CV melanoma cells, TRIM16 protein expression increased in a dose-dependent manner (Fig. 5A). We next assessed the effects of vemurafenib treatment on TRIM16 protein stability using cycloheximide (CHX) chase studies, and found that vemurafenib markedly increased TRIM16 protein stability in melanoma cells (Fig. 5B and Supplementary Fig. 4). To evaluate whether induction of TRIM16 expression was necessary for vemurafenib effects on melanoma cell viability we silenced TRIM16 gene expression using two TRIM16-specific siRNA's and treated BRAF-mutant A375 melanoma cells with vemurafenib. We found that both siRNAs blocked the reduction of cell viability induced by vemurafenib treatment (Fig. 5C). Lastly, we analyzed melanoma samples taken from patients prior to BRAF inhibitor therapy (N=9), during early treatment with vemurafenib (N=2, 960 mg twice daily) or dabrafenib (N=7, a total daily dose of ≥200 mg daily), and, at the point of clinical progression while still on the BRAF inhibitor (N=5). We showed that TRIM16 protein expression by IHC was significantly increased in patient melanoma tissues during early treatment with BRAF inhibitors (p=0.0018, two sided t-test, 95% confidence intervals 0.4 to 1.3) and reduced at relapse (Fig. 5D and 5E).

## DISCUSSION

Here we define an important role for TRIM16 expression in the early stages of melanomagenesis as an inhibitor of cell growth and migration through its effects on the melanoma cell expression of the inflammatory cytokine, IFNβ1. TRIM16 expression was markedly repressed in metastases compared with localized melanoma, suggesting that a low TRIM16 expression level in apparently localized melanoma may predict a high subsequent risk of distant metastases, thus our findings have significant diagnostic and therapeutic implications.

The mechanism by which TRIM16 expression is lost in melanoma cells is presently unknown. Although our data indicated multiple mechanisms, including promoter methylation, reduced protein stability and gene mutation, a much larger analysis of patient samples is needed. There are currently few good markers which can accurately predict the risk of metastases in patients with deeply invasive, but apparently localized, melanoma. We propose that TRIM16 expression is a good candidate for further evaluation as a prognostic marker in American Joint Committee on Cancer (AJCC) Stage II or III disease. Low TRIM16 expression in localized melanoma tissue may identify a patient cohort who will benefit from systemic therapy aimed at increasing TRIM16 levels and preventing metastasis.

Previous studies have indicated that the MAPK pathway plays a key role in melanoma cell migration [33]. ERK phosphorylation is a key mediator of cell proliferation and migration in metastatic melanoma. Interestingly, we found that treatment of melanoma cells with the BRAF inhibitor, vemurafenib, which attenuated ERK phosphorylation, resulted in increased levels of TRIM16 protein *in vitro* and *in vivo*. As TRIM16 is stabilized by vemurafenib at the protein level, it is possible that vemurafenib treatment may alter TRIM16 phosphorylation states or induce other post-translational modifications of TRIM16 which affect TRIM16 protein stability, as it has been shown previously that TRIM 16 undergoes serine-threonine phosphorylation and enhanced protein stability after retinoic acid treatment [28]. Moreover, we showed that the cytopathic effects of the successful BRAF inhibitor, vemurafenib, in part required induction of TRIM16 expression. This pattern was mirrored in melanoma tissue samples from patients treated with BRAF inhibitors, vemurafenib and dabrafenib, indicating that de-repression of TRIM16 expression in melanoma cells is a novel therapeutic approach and provides new insights into the mechanism of action of BRAF inhibitors.

**Figure 6:**
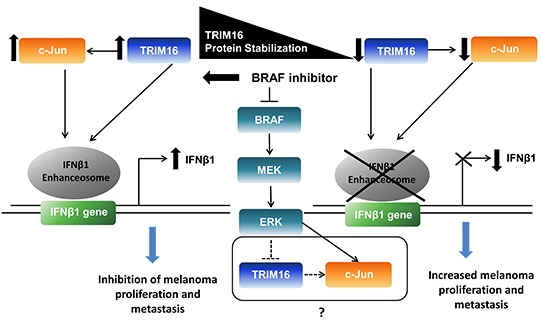
A proposed model for the role of TRIM16 in melanoma TRIM16 expression is significantly lost during progression from localized to metastatic melanoma. BRAF inhibitor increases and stabilizes TRIM16 protein levels. In the absence of BRAF inhibitor, TRIM16 and its target gene, c-Jun, are repressed and results in loss of IFNβ1 promoter binding and reduced enhanceosome complex activity and IFNβ1 transcription. BRAF inhibitor increases TRIM16 protein stability and consequently, IFNβ1 transcription. Inhibition of the BRAF/MEK/ERK signaling cascade may increase TRIM16, as TRIM16 may be a negatively regulated downstream target.

Our data has for the first time demonstrated a novel action of vemurafenib in suppressing melanoma cell growth in a TRIM16-dependent manner. We propose that TRIM16 suppresses melanoma cell growth via the upregulation of c-Jun and the formation of the enhanceosome promoter complex that is required for IFNβ1 gene transactivation (Fig. 6). As exogenous overexpression of IFNβ1 reduces cell proliferation and induces apoptosis in melanoma cells, restoration of endogenous IFNβ1 signaling by TRIM16 may enhance the anticancer activities of exogenous IFNβ1 [34]. Furthermore, combination treatment of recombinant human IFNβ and vemurafenib may reinforce each other and promote a co-operative anticancer signal.

## MATERIALS AND METHODS

### Tissue culture, plasmids and siRNA transfection

TRIM16-1 siRNA 5′AGTAATTCACCATGCA GGTTT-3′, TRIM16-2 siRNA 5′TCTCCCTCCTGC ATTTGTGTT-3′ were custom designed and transfected at 20nM using lipofectamine 2000 as the transfection agent and siControl non-targeting pool (Thermo Scientific, Waltham, MA, USA) as a control. Melanoma cell lines, A375 and G361 were purchased from ATCC. Lines, CHL-1, IPC-298, and SK-Mel2 were kindly gifted from Professor Grant MacArthur at the Peter MacCallum Institute, Melbourne. Lines, Mel-JD, Mel-RM, Mel-CV, M4405 and MM200 were kindly gifted from Professor Xu Dong Zhang at the University of Newcastle. All lines were cultured in Dulbecco's modified eagle medium (Life Technologies Australia, VIC, Australia) supplemented with 5% foetal calf serum and incubated at 37°C/5% CO_2_. TRIM16 over-expression was achieved by transient transfection of the pcDNA3.1/myc-his tag plasmid containing the full-length TRIM16 cDNA under a CMV promoter using lipofectamine 2000 (Life Technologies Australia, VIC, Australia).

### Cyclohexamide, 5-aza-2′-deoxycytidine (5-Aza), MG-132 and vemurafenib treatments

For the cycloheximide (CHX) chase assays, A375 and Mel-CV cells were seeded at a density of 1×10^4^ cells/6 well plate and cultured overnight. Baseline samples were taken in the absence of CHX and CHX (with our without 0.5 μM vemurafenib (Selleck chemicals, TX, USA)) applied at 100μg/mL with lysates prepared at 2, 4, 6, 8, 14 and 24 hours for western blot analysis using TRIM16 and GAPDH specific antibodies. For the 5-aza treatment, cells were seeded at 1×10^6^ cells in T-75 flasks and the following day, 30 μM of 5-Aza was applied and cultured for 24 hours (harvested for RNA) and 72 hours for protein lysates. For the inhibition of protein synthesis, cells were treated with 30 μM MG-132 (Sigma St Louis, MO, USA) for 8 and 16 hours when whole cell lysates were prepared. For the TRIM16 gene silencing studies, vemurafenib was applied at 0.5μM in the presence of control or TRIM16 specific siRNAs and allowed to culture for 72 hours before cell viability was determined.

### Scratch wound healing and transwell assay

For the scratch assay, G361 cells were seeded at 1×10^5^cells/6 well plate and allowed to culture overnight. Cells were transiently transfected with pcDNA3.1 expressing empty vector or TRIM16. After 24 hours transfection, a scratch wound was applied using a pipette tip and a baseline image obtained. Scratch wound closure was monitored over a period of 24 hours. For the transwell assay, NHEM cells were seeded at 2×10^4^cells/transwell chamber (Life Technologies Australia, VIC, Australia) in a cell suspension of 20 nM siTRIM16-1, siTRIM16-2 or siControl. After 5 hours transfection, the transfection complexes were removed and melanocyte media placed into the upper chamber. Conditioned media was placed in the lower chamber as a chemo-attractant. Melanocytes were allowed to migrate for 48 hours before the chamber was fixed and stained for cell counting.

### Chromatin immunoprecipitation assay

Chromatin immunoprecipitation was performed using the Millipore ChIP kit (Merck/Millipore, Germany) with modification to manufacturer's instruction. G361 cells were sonicated for 45 min using high sonication and 45 min low sonication in the DNA fragmentation step. Specific antibodies for TRIM16 (Bethyl, TX, USA), c-Jun (Cell Signaling, Massachusetts, USA catalogue #9165) and IgG (Dako, Victoria, Australia) were used for respective protein pull-down. DNA from the pull-down was purified using the MiniElute PCR purification kit in accordance with the manufacturer's instructions (Qiagen, VIC, Australia). PCR protocol was as follows: 95C: 2min/95C:45 sec, 58C:45 sec, 72C:45 sec/72C:7 min (x35 cycles). Primer pairs were as follows: IFNβ1 promoter (forward 5′-AGGTCGTTTGCTTTCCTTTGC-3′, reverse 5′-GACAACACGAACAGTGTCGC-3′) with negative control primers (forward 5′ACTGCCTGCATTAAGGGCAA-3′, reverse 5′-ACAGAAGGCCTCATCACTGC-3′).

### Tissue microarray construction

Reference sections of the donor tissue block were cut, H&E stains performed and the slides marked with a 1mm circle to identify areas of tumor. Triplicate tissue cores 1 mm in diameter were taken from the donor paraffin block using the marked section as a reference and then arranged in a blank paraffin block by a MTA-1 Manual Tissue Arrayer (Beecher Instruments, Sun Prairie, WI, USA). Once constructed, the microarrays were baked for 30 minutes at 37° C on a glass slide to even out the surface and fuse the paraffin in the cores and donor block.

### Immunohistochemistry of patient tissue samples

Following Human Research Ethics Committee approval (Protocol X11-0023 & HREC/11/RPAH/32 and Protocol X10-0300 & HREC/10/RPAH/530), paraffin-embedded tissue blocks of excised human skin specimens were retrieved from the Department of Tissue Pathology and Diagnostic Oncology at the Royal Prince Alfred Hospital, Sydney, Australia. Whole tissue sections from compound and dysplastic nevi, *in situ* melanoma, dermal invasive melanoma, lymph node metastases and distant metastases were probed with a specific TRIM16 custom made antibody at 1:500 dilution (Biosource, CA, USA) and IgG at 1:500 dilution (Dako, VIC, Australia) was used as a negative control. A rabbit-biotinylated secondary antibody was used at 1:500 dilution followed by streptavidin-HRP (Dako, VIC, Australia) and stained by ACE peroxidase substrate (Vector Laboratories, CA, USA). Samples were counter-stained with Haematoxylin solution (Sigma-Aldrich, NSW, Australia). Tissue microarrays of 170 patient lymph node metastasis samples (in triplicate) were stained as described above. Samples were graded independently by two observers, who were blinded to patient outcome, using a semi-quantitative scale of 0-4, with 4 being the highest staining intensity and 0 as negative staining. Tissues were graded at two different sites averaging 150 cells/field to allow for heterogeneity of staining intensity within the tissue [35]. The cohort was divided into ‘high’ and ‘low’ staining groups based on the median value cut off and significance were determined by applying the log rank test.

### Protein extraction and western blotting

Cell lysates were prepared using RIPA buffer and protein concentration were determined in cleared lysates using the Pierce BCA method (Thermoscientific, Rockford, IL, USA). Protein lysate was standardized and run on Criterion Tris-HCl 10.5-14% gels (Bio-Rad, NSW, Australia). Western blotting used the following antibodies: polyclonal TRIM16 (Bethyl laboratories, TX, USA), anti-myc tag antibody (Cell Signalling Technology, Danvers, MA, USA), rabbit polyclonal actin antibody (Sigma, St Louis, MO, USA) and anti-GAPDH antibody (Abcam, NSW, Australia).

### PCR array and quantitative real-time polymerase chain reaction (RT-qPCR)

RNA was extracted from TRIM16 transfected G361 cell lines using the PureLink RNA mini extraction kit (Ambion, VIC, Australia), in accordance with the manufacturer's instruction. Cancer PathwayFinder PCR array was performed according to the manufacturer's instruction (SABiosciences, PAHS-033A) using an ABI Prism 7900 sequence detection system (Applied Biosystems, VIC, Australia).

### PCR of genomic DNA and sequencing

Genomic DNA was extracted using TRIreagent (Sigma-Aldrich, NSW, Australia) in accordance with the manufacturer's instruction. PCR of six TRIM16 exons was performed using the following primer pairs: Exon 1 Forward (F) 5′-TGGCCGAGCTTCC TCTGGGA-3′, Reverse (R) 5′-ATGAATGGTCCCCAAGCACTCAC3′. Exon 2 F 5′-GTGCATTGGGCTCCTTCCTCCTTA-3′, R 5′-CTGGCTGACCCAGGCTGGTCTT-3′. Exon 3 F 5′-TTGGCTGCCTTCCACCCCCA-3′, R 5′-TGG GGCAGCTGTGGGATGCC-3′. Exon 4 F 5′-GCA AGTTCTGCTGTTTCCTTTTCTGC-3′, R 5′-CACTA TAGTCCATG GCCCAGAATGC-3′. Exon 5 F 5′-CTGGACAGGTGTGCATTCACAACTC-3′, R 5′ ACTG ATATCAACAACTGGAGAAGCGG-3′. Exon 6 F 5′ TCT CCTGCCTTCTGTGTCTCCTCA G-3′, R 5′-AGCCA GCTACCATCAGCAGTTATTTC-3′. PCR product clean-up was performed using ExoSAP-IT (Affymetrix, CA, USA). Sequencing was performed at the Australian Genome Research Facility (Westmead, NSW, Australia) using BigDye Terminator sequencing (Life Technologies Australia, VIC, Australia).

### Statistical analysis

Data were analysed using the student's t-test or log-rank test where appropriate for the analysis. Results were considered statistically significant with a p value of <0.05. Statistical analysis was performed using GraphPad Prism version 6.01 (GraphPad software Inc, La Jolla, CA). All statistical tests were two sided. The Kaplan Meier overall survival curve was based on ‘high’ (≥1) or ‘low’ (<1) staining from the patient median value of 0.995 (arbitrary units). The p value was determined by the log-rank test between high and low groups.

## SUPPLEMENTARY FIGURES


