# Supplementary material for: TRIM16 inhibits proliferation and migration through regulation of interferon beta 1 in melanoma cells
Source: Oncotarget. 2014 Nov 3;5(20):10127–39. doi: 10.18632/oncotarget.2466 (PMC4259410; doi:10.18632/oncotarget.2466)
Supplement: Supplementary file 1 [file oncotarget-05-10127-s001.pdf]

SUPPLEMENTARY FIGURES

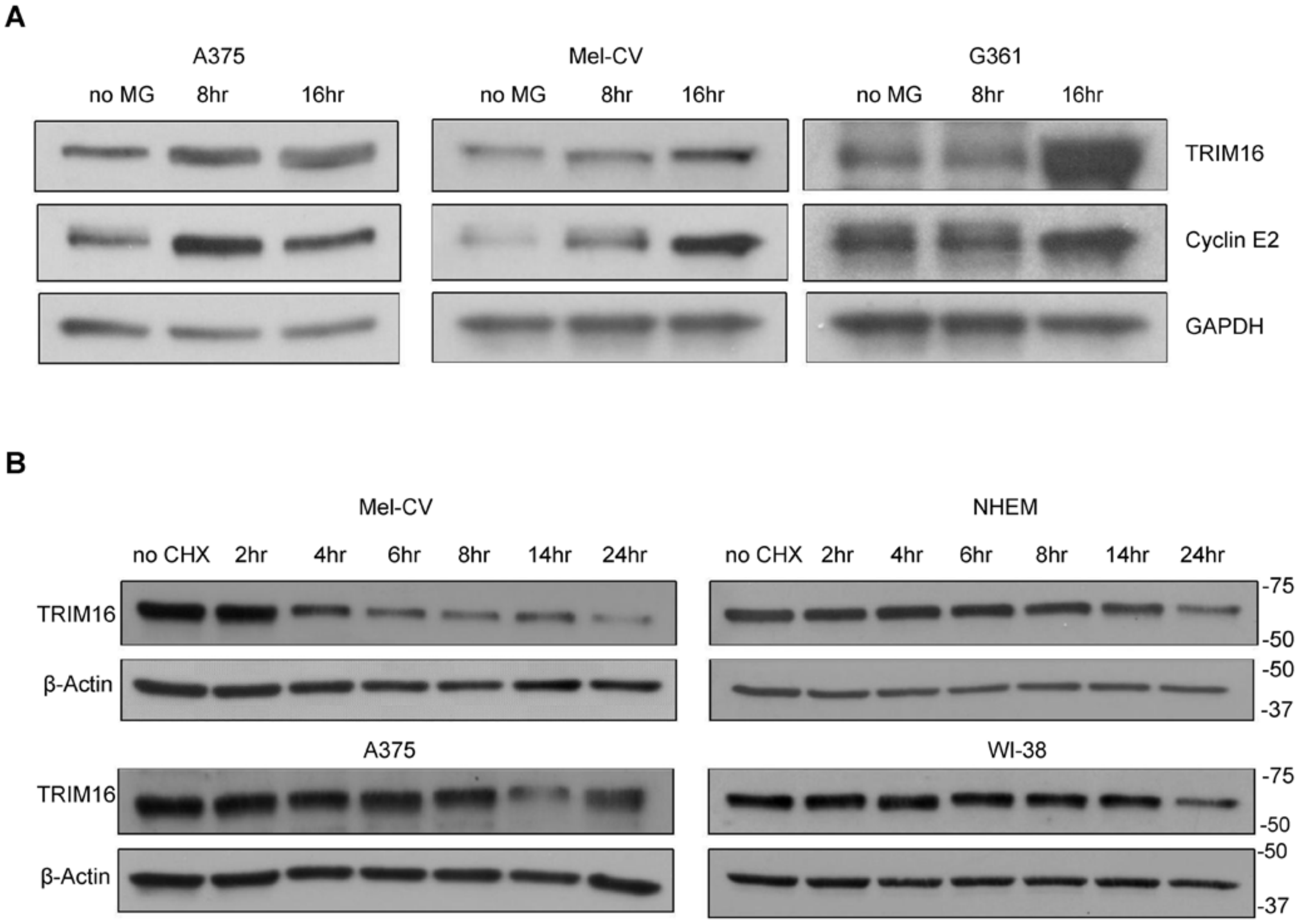

Supplementary Figure S1: (A) The proteasome inhibitor MG-132 increased TRIM16 protein levels in melanoma cells. Melanoma cells were treated with 30µM MG-132 for 8 and 16 hours. Whole cell lysates were prepared and Western blots were probed with anti-TRIM16, & anti-cyclin E2, and anti-β-actin antibodies as positive and loading controls. (B) TRIM16 protein half-life was analysed by Western blotting in melanoma cell lines (Mel-CV, A375), NHEM and WI-38 human fibroblasts following CHX treatment at a final concentration of 100 µg/ml over 24 hours. At the specified time points, the cells were harvested and total cellular protein was extracted for Western blotting.

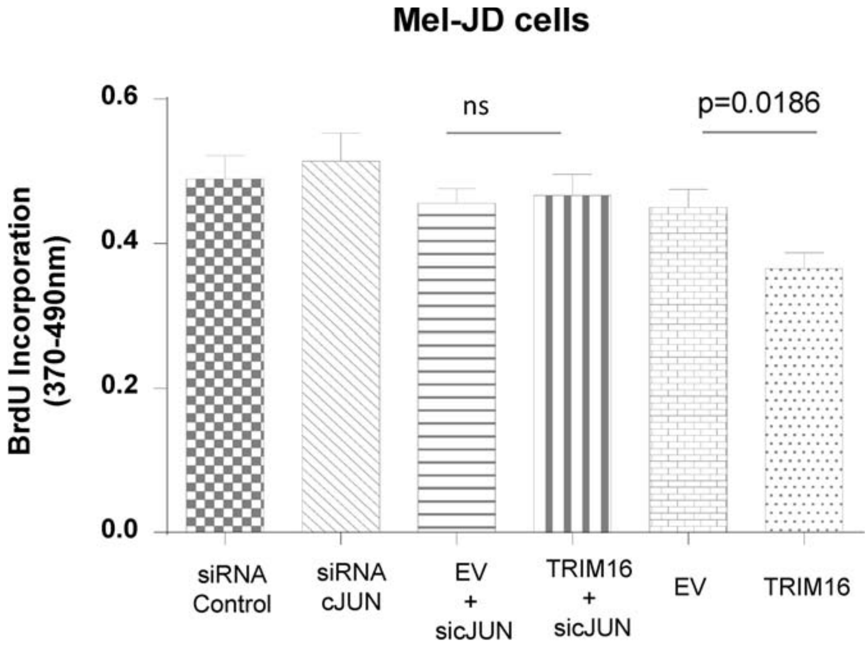

Supplementary Figure S2: c-Jun expression is required for TRIM16-mediated inhibition of cell proliferation. Mel-JD cells were transiently transfected with either control siRNA, c-Jun siRNA, EV + sic-Jun, TRIM16 expression vector + sic-Jun, EV, or TRIM16 expression vector for 24 hours. Cell proliferation was measured by the BrdU incorporation assay in three independent experiments.

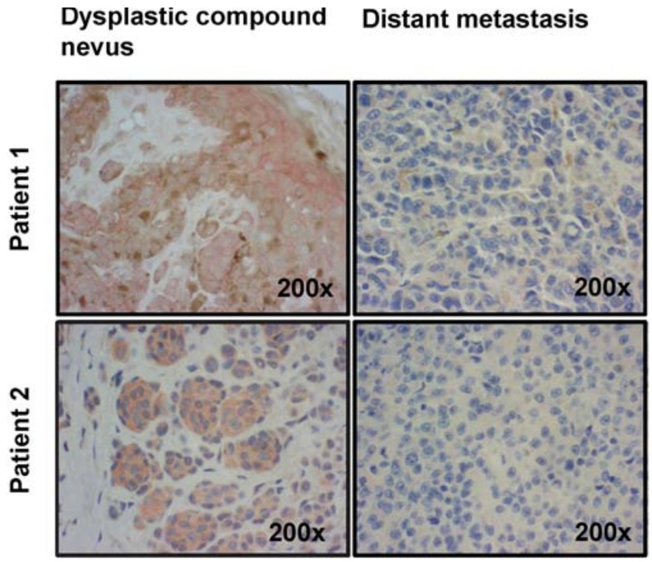

Supplementary Figure S3: Representative immunohistochemical staining for IFNβ1 expression for two dysplastic compound nevi and two distant metastases of patient samples.

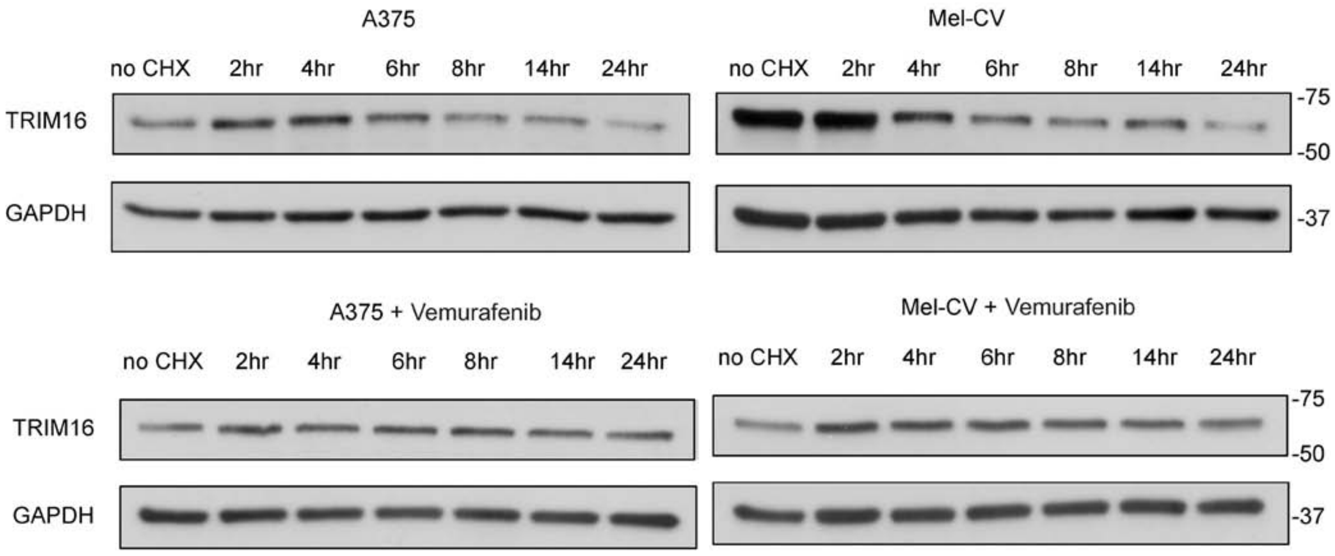

Supplementary Figure S4: TRIM16 protein stability was assessed in melanoma (A375 and Mel-CV) cells following treatment with vemurafenib at 0.5 and 1.5µM respectively, and CHX at 100µg/mL. At the specified time points, the cells were harvested and the protein was extracted for analysis by Western blotting against anti-TRIM16 or anti-β-actin antibodies.
